# iHandU: A Novel Quantitative Wrist Rigidity Evaluation Device for Deep Brain Stimulation Surgery

**DOI:** 10.3390/s20020331

**Published:** 2020-01-07

**Authors:** Elodie Múrias Lopes, Maria do Carmo Vilas-Boas, Duarte Dias, Maria José Rosas, Rui Vaz, João Paulo Silva Cunha

**Affiliations:** 1 INESC TEC and Faculty of Engineering, University of Porto, 4099-001 Porto, Portugal; elodie.murias2@gmail.com (E.M.L.); carmo.vilas.boas@inesctec.pt (M.d.C.V.-B.); duarte.f.dias@inesctec.pt (D.D.); 2 Centro Hospitalar do Porto, Hospital Santo António, Unidade Corino de Andrade, E.P.E., 4099-001 Porto, Portugal; 3 Department of Neurology & Movement Disorders and Functional Surgery Unit of Centro Hospitalar Universitário São João, E.P.E., 4099-001 Porto, Portugal; rosas.mariajose@gmail.com (M.J.R.); ruimcvaz@gmail.com (R.V.); 4 Department of Clinical Neurosciences and Mental Health, Faculty of Medicine University of Porto, 4099-001 Porto, Portugal; 5 Clinical Neuroscience Centre, Hospital CUF Porto, 4099-001 Porto, Portugal

**Keywords:** parkinson’s disease, deep brain stimulation, wrist rigidity, intra-op classification, wearable system, inertial sensors, mathematical models

## Abstract

Deep brain stimulation (DBS) surgery is the gold standard therapeutic intervention in Parkinson’s disease (PD) with motor complications, notwithstanding drug therapy. In the intraoperative evaluation of DBS’s efficacy, neurologists impose a passive wrist flexion movement and qualitatively describe the perceived decrease in rigidity under different stimulation parameters and electrode positions. To tackle this subjectivity, we designed a wearable device to quantitatively evaluate the wrist rigidity changes during the neurosurgery procedure, supporting physicians in decision-making when setting the stimulation parameters and reducing surgery time. This system comprises a gyroscope sensor embedded in a textile band for patient’s hand, communicating to a smartphone via Bluetooth and has been evaluated on three datasets, showing an average accuracy of 80%. In this work, we present a system that has seen four iterations since 2015, improving on accuracy, usability and reliability. We aim to review the work done so far, outlining the iHandU system evolution, as well as the main challenges, lessons learned, and future steps to improve it. We also introduce the last version (iHandU 4.0), currently used in DBS surgeries at São João Hospital in Portugal.

## 1. Introduction

Parkinson’s Disease (PD) is a neurodegenerative disorder caused by a dopaminergic neural loss in the substantia nigra. Dopamine has an inhibitory effect on the excitatory signals that are propagated from the nigrostriatal pathway to the motor cortex. Consequently, the decrease of dopamine transmission between neurons will lead the motor pathway to remain in an excited state. The cardinal symptoms of PD include the bradykinesia, resting tremor, postural instability, and rigidity [1].

Nowadays, the cure for PD is still unknown and dopamine replacement therapy with Levodopa (L-dopa) remains the major medical approach to control the PD symptoms. However, over the years, the use of L-dopa may lead to highly disabling fluctuations and dyskinesias [2]. More recently, deep brain stimulation (DBS) surgery has emerged as the second therapy in those patients, leading to the improvement of dyskinesia, as well as of dopamine-responsive motor symptoms [3]. The DBS surgical procedure is based on the implantation of stimulation electrodes at high frequencies (130 Hz) with a pulse length at 60 μs in the basal ganglia structures (subthalamic nucleus (STN) or internal globulus pallidum (GPi)), aiming to promote the functional inhibition of the excited motor control pathways [3]. The stereotactic target of stimulation is first defined based on pre-operative medical imaging. Then, an electrophysiological exploration is performed with a tetrapolar electrode, while varying the stimulation parameters (current intensity, voltage, depth and anatomical position) and testing symptoms and side effects to determine the final electrode configuration [3].

To assess the improvement of patient response to a therapeutic approach, the severity of the PD symptoms may be evaluated. Rigidity is present in 90–99% of patients [4]. To assess the rigidity improvement, the clinician usually imposes a passive flexion to a patient’s joint and attributes it a subjective score, translating the severity of the muscles rigidity, ranging from 0% to 80% of improvement [3]. The wrist rigidity is a trustworthy functional feature since the cogwheel rigidity, which is defined as a PD-typical muscular stiffness throughout the range of wrist passive movement, can be easily perceived [5]. Generally, a second neurologist performs an intra-operatory blind test to confirm the first evaluation. Nevertheless, this assessment is subjective and depends on the experience and perception of each neurologist. Therefore, it is crucial to develop quantitative methods for assessing rigidity. Table 1 presents a summary of the literature in this issue.

Patrick et al. initiated the first attempts to evaluate the rigidity of the elbow joint as a measure of mechanical impedance. For this purpose, they developed a custom-made device which includes two pads placed where the physician will force the joint flexion, measuring the resultant force applied on them, and a gyroscope sensor which measured the angular velocity [6]. Later, Shapiro et al., showed the effectiveness of DBS stimulation by measuring the resistive torque of the elbow joint movement. The proposed methodology allowed the authors to discriminate PD patients from healthy controls [7]. Following these studies, other biomechanical variables have been proposed for rigidity quantification, including the peak torque [8], impulse [11], difference of bias [9], elastic stiffness [11], damping constant [11], and surface electromyography of the biceps and triceps branchii [9,10]. Despite the advances made, the proposed systems were bulky and some of them provided values of features that could hinder the intuitive understanding of the physician about the rigidity severity. Further, these methodologies relied on relatively heavy processing which might lead to delays in the output, hampering its immediate feedback to physicians when searching for the best DBS electrode positioning and parameters during surgery. Therefore, they were not considered suitable for intra-operative conditions, such as DBS.

In 2014, Kwon et al. were the first to evaluate the wrist rigidity during intraoperative DBS, measuring several biomechanical properties, such as mechanical impedance, impulse, elastic stiffness and damping constant. The rigidity evaluation device consisted of a potentiometer to measure the joint angle, a load cell and an accelerometer to measure inertia [11]. Later, Shah et al. developed a method to measure rigidity during DBS using an accelerometer sensor. The authors concluded that a change in rigidity modifies the speed of passive movements [14]. Van den Noort et al. conducted a proof-of-principle study in which the patient wrist rigidity was assessed using a custom-made system, the PowerGlobe, which included inertial and force sensors [16]. Similarly, Angeles et al. used inertial motion, muscle activity and force sensors to measure both elbow and wrist rigidity to assess the impact of DBS therapy on the rigidity symptom [17]. Di Biase et al., on the other hand, measured elbow and wrist rigidity and their changes during DBS procedure, using magnetic-inertial wearable sensors [18]. More recently, Perera et al. developed a new system to measure the rigidity of the metacarpophalangeal joint, consisting of a miniature motor to flex the third digit of the hand. With the developed device, the authors studied the impact of the DBS and performed comparisons between PD patients and healthy individuals [20]. Despite these advances in quantifying rigidity reduction during DBS, none of these systems has been able to provide real-time feedback of the patient’s rigidity to the examiner during DBS surgery procedure, supporting the decision-making towards the final placement of the DBS electrode.

Our group has developed a real-time feedback system to evaluate the patient’s wrist rigidity in situ. To our knowledge, there is no other group following this line of research. In [13], we presented the iHandU system: a novel, comfortable and wireless system, designed to classify the wrist rigidity during the DBS, by deriving features from angular velocity values of wrist flexion and using a polynomial mathematical model to classify signals, stored in a computer. This system was able to provide in real-time the wrist rigidity reduction the DBS procedure to clinicians, hence supporting the physician decision. We showed that the iHandU classified correctly 83.9% of the evaluated signals against the blind agreement of two experts. The main difficulties were found in distinguishing intermediate levels of wrist rigidity. Also, we suggested that the wearable device should be improved and adapted to the operating room environment. In [15] and [19], we initiated efforts to improve the iHandU system at three different levels: wrist rigidity classification model, system’s hardware and system’s software. This work gave rise to three new versions along the years: the iHandU 2.0, described in [15], the iHandU 3.0, described in [19] and our more recent version, the iHandU 4.0, which was developed to increase system comfort at the patient and clinician levels, as well as to enable faster signal processing and an easier methodology to record surgery-related information. 

This publication aims to review all the work done so far, outlining the iHandU system evaluation over these years, with a special focus on the wrist rigidity models developed to enhance the iHandU performance and drawing the lessons learned from each iteration. We also introduce the iHandU 4.0, which is currently being use in all DBS surgeries performed at São João Hospital in Porto, Portugal. 

This paper is organized as the follows. In Section 2 (iHandU 1.0-Proof-of-Concept) we describe the methodology, results and limitations of the first iHandU version. In Section 3 (iHandU System Evaluation), we describe the following versions of iHandU system in terms of hardware, software and wrist rigidity classification models, while in Section 4 (Wrist Rigidity Classification Models), we address the classification models developed over time in greater detail. In the last section (Conclusions and Future Directions), we emphasize the conclusions of this research line and what we have learned throughout each iHandU version, as well as what we intend to achieve in the near-future.

## 2. iHandU 1.0-Proof-of-Concept

**Figure 1:**
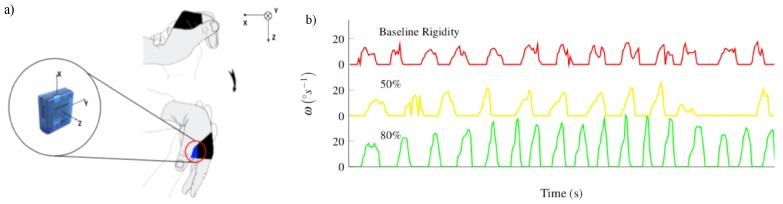
(**a**) Setup of the motion sensor, which is placed on the patient’s hand, during the passive wrist flexion. This configuration ensures that the wrist flexion is performed along the MoMo system, independently from the hand pose. Extracted from [13]; (**b**) Example of angular velocity signals correspondent to when baseline rigidity was being assessed, to 50% and 80% improvement in rigidity. Each arcade corresponds to a wrist flexion. With a reduction of rigidity, it is clear that the signal becomes smoother and higher peak values are achieved.

The general architecture of the iHandU system includes a Motion Mote (MoMo), consisting in a Bluetooth-enabled Inertial Measurement Unit (IMU) embedded into a specially designed textile band, that is worn on the palm of the patient’s hand. This sensor communicates (via Bluetooth v2.0) with an external device for data analysis and presentation. Figure 1 shows the existing setup of the motion sensor and angular velocity signals, acquired from different rigidity states. This device was developed a few years ago by our research group and has a gyroscope (± 2000°/s) an accelerometer (2, 4, or 8 g) and a magnetometer (with compass heading accuracy of 1° to 2°) with a battery for up to 8 hours of continuous working. A dedicated microcontroller unit (MCU) collects data from all the sensors at a sampling rate of 42 Hz and then transmits the data via Bluetooth to an external device wirelessly [13]. In this first proof-of-concept version, the MoMo was transmitting data to an Intel Core i7-4600 CPU @ 2.70 GHz computer, which allowed data visualization and computation of the reduction of wrist rigidity during DBS surgery, through a National Instruments LabView 2014 script [13]. This data processing was performed using only the data of the y-axis from gyroscope sensor, due to its high level of pronunciation during the wrist passive flexion. Such a configuration was designed with the aim to not interfere in the normal passive wrist flexion movement nor with the surgical procedure.

### 2.1. Subjects and Experiments

The system was used during the bilateral subthalamic nucleus (STN) DBS of parkinsonian patients, while an examiner was evaluating the wrist rigidity improvement for different stimulation settings. The subjects had their medication withdrawn 12 h before the procedure and were under local anaesthesia during the surgical procedure. A stereotactic target for stimulation and trajectory for the electrode were previously defined, based on medical imaging. Then, electrophysiological exploration was performed during the surgical procedure to determine the optimal electrode placement. The stimulation frequency was set at 130 Hz, while voltage, depth and placement were varied. To train the rigidity classification model, data was first acquired from a total of 6 patients (48 signals); then, 4 patients were considered (156 signals) to test them [13]. The wrist rigidity improvement classification derived by our system was compared to the evaluation of at least two experts’ agreement. A classification was considered accurate if it was within a range of 5% away from their classification. Patient monitoring was authorized by the local ethics committee and all patients signed an informed consent form.

### 2.2. Signal Processing

The data acquired from the y-gyroscope sensor (*y*) was first converted to angular velocity based on the gyroscope specifications as follows:

(1) $$\omega_{y}=\frac{y}{2^{15}}\;2000\;\left({}^{\circ}\;s^{-1}\right)$$

Then, a 4-sample moving average low-pass filter was applied for non-movement noise reduction. To keep only samples corresponding to wrist flexion movements, only the negative arcades of ω were considered. Note that the MoMo orientation makes the flexion movement to be in the opposite direction of the positive rotation around the *y*-axis. For every 300 samples of angular velocity, two angular velocity features were extracted: the average angular velocity, $\mu_{\omega_{y}}$, and the average peak value, $\mu_{p_{y}}$. Absolute peaks were computed as the highest values between two valleys of the signal, within a margin of 0.2 °s^−1^. Although low rigidity and $\mu_{p_{y}}$ values have a direct correlation to wrist rigidity states (see Table 2), this feature, by itself, did not guarantee accurate discrimination of rigid *versus* non-rigid, since signals with widely different shapes can present similar peak values. Elongated signal arcades, few peaks in a certain period of time or unexpected plateaus during the flexion movement can by other hand correspond to some residual rigidity even in signals with high amplitude and must be taken in consideration. Such information can be yielded by $\mu_{\omega_{y}}$, since the average of the signal decreases for non-smooth signals. Therefore, we proposed to combine these two features by deriving a signal descriptor [13], defined as:

(2) $$\varphi_{y}=\sqrt{\mu_{\omega_{y}}.\mu_{p_{y}}}$$

The signal descriptor aimed at discriminating between the stimulation settings that improve the patient’s condition and those who do not. We then clustered the training dataset into the specified classes of rigidity improvement: 10%, 20%, 30%, 40%, 50%, 60%, 70%, and 80%, according to the clinician’s classification of the wrist rigidity improvement. Jarque–Bera tests [21] confirmed the data normality profile and descriptive power was assessed using two-tailed t-tests [13].

### 2.3. Classification Model

The signal descriptor defined by the Equation (1) was used to build the wrist rigidity model, where the rigidity during passive wrist flexion was labeled according to the expert’s assessment (medical label) following a discrete decimal scale ranging from 0% to 80% of rigidity improvement. Then, we computed the average values of the signal descriptors belonging to the same medical label classes. Finally, we determined the polynomial mathematical model that best approximates the modeled relationship between the percentage of rigidity improvement (rigidity label) and the signal descriptor. Approximations of higher degrees were discarded since they can lead to overfitting and can be less responsive to widely different incoming signals. Standard machine learning techniques were also excluded due to their heavy computational cost, which would limit the implementation of the classification model with local signal processing in “wearable-embedded” signal processing hardware. We used a custom-made program running on MathWorks^®^ MATLAB to analyze the training set. The training error was estimated using the leave-one-out cross-validation with 5000 iterations. The classification model was tested in four patients, during DBS surgery [13].

### 2.4. First Model Usability Results

We designed a system to quantitatively assess the wrist rigidity during DBS procedure, capable to send the result to clinicians in real-time, helping in the interactive determination of the optimal stimulation setting.

**Figure 2:**
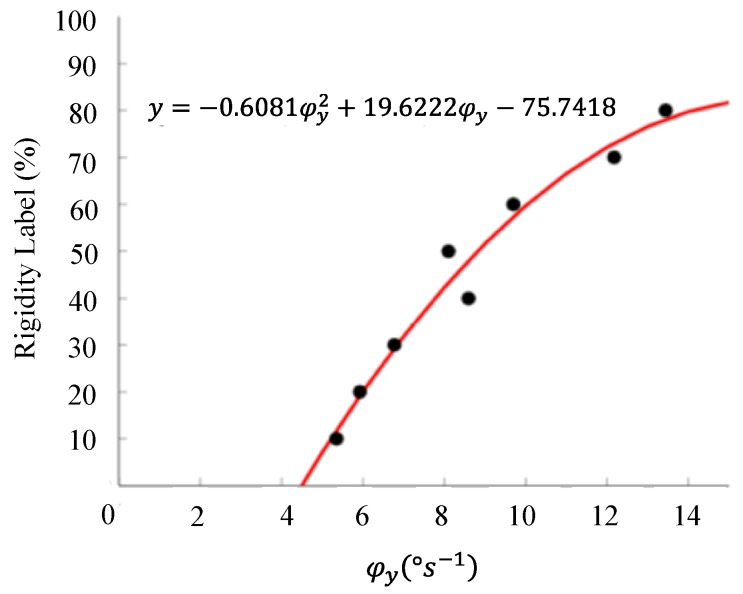
The polynomial function that best modeled the relationship between the percentage of wrist rigidity improvement (Rigidity Label) and the signal descriptor. Adapted from [13].

From the statistical analysis’ results summarized in Table 2, we concluded that $\varphi_{y}$ had more discriminative power between rigid and non-rigid states, since their p-values are lower than the p-values of its counterparts. The polynomial model derived for rigidity classification is presented in Figure 2 and had a training error of 8.24±7.95%. This error range was considered acceptable, since we modeled a discrete scale using a continuous function. The classification model allowed a correct classification of 131 out of 156 classifications of rigidity, corresponding to an accuracy of 83.9% [13].

The main difficulties were found when distinguishing intermediate levels of wrist rigidity, whose correlation with the classification model were lower. This could be related to the nonlinear dependence of the rigidity with the angular velocity amplitude. Therefore, we hypothesized that this model could be improved by considering other angular velocity features, in addition to the existing ones, such as the cogwheel rigidity of wrist and the patient’s rigidity baseline, i.e., the Unified Parkinson’s Disease Rating Scale (UPDRS) scores before starting stimulation.

The premise of this analysis is that the assessment is being performed correctly, ensuring three main conditions: (1) It is expected that the imposing flexion should be the largest amplitude that occurs on the y direction, avoiding rotational movements; (2) The force applied by the clinician does not vary greatly over the course of DBS surgery and between surgeries as well; (3) Patients can not actively try to counter or help in bending the wrist, in order to avoid undesired rigidity profiles. The last requirement can be partially fulfilled in our system, since we can label these signals as “invalid” after indication of the clinicians. The first and second requirements, on the other hand, cannot be secured in our system, which presents another limitation. Therefore, we also concluded that the angular velocity acquired from other gyroscope axes (besides the y axis) should be explored, as well as the possibility of integrating force sensors into our system. Another limitation of the system arises due the angular velocity features of the training set which were extracted from every 300 samples, while the system classified every train of 200 samples (~4 s). This might have influenced the classification as it might lead to a low average angular velocity. Therefore, we suggested to decrease the number of samples to extract the angular velocity features.

In addition to the limitations on the classification model, the first version also had limitations on the hardware level. The iHandU 1.0 required connection to a computer for signal processing and data visualization. Consequently, to provide the wrist rigidity improvement to clinicians at each stimulation, i.e., in real-time, it was necessary to integrate the computer into the operating room environment. This was a disadvantage due to the space that a computer occupies. Therefore, we suggested that a smaller external device (but with equivalent processing power) should be integrated to fulfill that role, improving usability in intra-operatory conditions. Also, we agreed that the wearable device could be improved in term of patient’s comfort and clinicians’ usability, since this version was large, thicker and difficult to fix to the patient’s hand.

In this section we have presented a first proof-of-concept of the iHandU system, reported in [13], which showed good results. After this first iteration, and bearing in mind the limitations presented, the iHandU system has been improved at different levels: (1) design, implementation and test of four different new wrist rigidity classification models (Section 3.1 and Section 4); (2) wearable device, by adapting it for operating room environment (Section 3.2); (3) smartphone application, user-friendly, capable to store, process and show real-time results during intra-operatory assessment (Section 3.3). This was an iterative process where several versions were developed and validated in real DBS surgeries to improve the iHandU usability and functionality in the operating room. The following sections present and discuss these enhancements, showing their relevance and impact, such the methodologies used and validation results. It is of major importance to mention that after this technology proof-of-concept, a patent was filled covering the use of these type of devices to assess rigidity in DBS surgeries, which is currently covered in Europe Union, United States, People’s Republic of China, Canada, Japan and Republic of Korea, with the number WO2016166702A1.

## 3. iHandU System Evolution

Since the first version, described in [13], the iHandU system has undergone several developments in terms of software, hardware and classification model, with the aim of improving the system performance, as well as making it more suitable for intra-operative conditions and more comfortable for both patients and clinicians. Table 3 summarizes the iHandU evolution with the added features and new developments of each consecutive version. Each system version was developed with the aim to overcome some limitations underlying the previous version. Note that the wrist rigidity classification models of iHandU 2.0 and 3.0 were already described in scientific publications (in [15,19], respectively). Here, on the other hand, we focus on the evolution of the system at three levels (Table 3), and we present the most recent version that is currently being used: the iHandU 4.0.

### 3.1. Wrist Rigidity Classification Model Evaluation

The first iHandU classification model, the y-Gyroscope Model (yGM) [13], presented in the Section 2, labeled correctly 83.9% of the evaluated signals against the blind-agreement of two specialists. The main limitations were found when classifying intermediate levels of rigidity. It was concluded that this could be related to the nonlinear dependence of the rigidity with the angular velocity amplitude. Therefore, we suggested that the system performance could be improved by considering other features besides used ones (angular velocity average and mean of peak values). Other limitations found was the fact that velocity was dependent of the axis and the force exerted by the clinician during the passive flexion exam, requiring the clinician to apply approximately the same force and in the same direction in every trial. Also, we defended that the extraction of the angular velocity features for every 300 samples, although classification is made with 200 samples, may lead to a low average angular velocity, which could decrease the system performance.

Our group has been trying to overcome the limitations of the first model since 2015, which resulted in two scientific publications. First, in 2016, Assis et al. attempt to increase the system performance by including the patient’s baseline wrist rigidity information in the classification model. This approach was designated as the multi-model (MM) [15], consisting of two rigidity models, specific for each baseline rigidity: low or high rigidity states, measured before stimulation. The most appropriate model can then be selected at start-off of the surgery, according to the physician’s initial assessment. Despite the efforts, the authors concluded that the MM did not improve the system performance.

Later, in 2018, Lopes et al. developed the cogwheel rigidity model (CRM), in which the number of cogwheel rigidity artefacts was included in the signal descriptor as a third angular velocity feature. To detect the cogwheel rigidity artefacts, the authors used the methodology developed by [13]. Simultaneously, the same authors developed the three-gyroscope-axis model (3GAM), in which the angular velocity of the three Cartesian axes were included in the classification model, in order to solve the axis-dependence limitation of the system. Despite the efforts made, the authors also concluded that these two models did not improve the first system performance [19].

On the other hand, in both studies, the authors have proven that the yGM remains the best model to classify the wrist rigidity improvement, and a similar accuracy value of 80% was achieved (as it was also shown in the first model [13]). This result proves the robustness of this rigidity classification model. In addition, in [19], the authors also reduced the number of samples to extract the angular velocity features of the yGM to 200, instead of 300. Despite this approach (designated as y-Reduced Model (yRM) did not improve nor decreased the system performance, it enables a faster calculation of wrist rigidity improvement during the surgical procedure, reducing in this way the overall classification procedure duration during DBS. 

Note that each system version was developed with the aim to overcome some limitations underlying the previous version. In order to do this, it was necessary to collect more data containing new sensing features necessary for each classification model. For example, to develop the MM, it was necessary to record the UPDRS data, which was not considered in the iHandU 1.0. Also, to develop the 3GAM, as well as the CRM, it was necessary to update the iHandU version in order to save the angular velocity data of the three-gyroscope-axis and the cogwheel rigidity artefacts number, respectively. Consequently, these iterations were developed at different times with different features which lead to the analysis of distinct datasets. 

Currently, the yRM is the wrist rigidity classification model used during DBS surgeries. These models will be discussed in more detail in the Section 4.

### 3.2. Wearable Device Evolution

**Figure 3:**
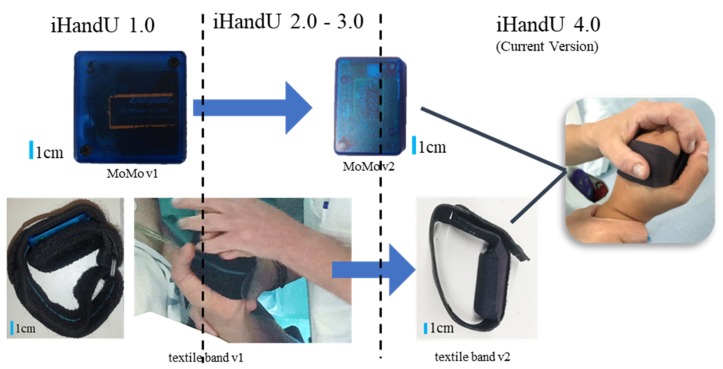
Evolution of the iHandU system wearable device. The general architecture of the iHandU system includes a Motion Mote (MoMo): a Bluetooth-enabled Inertial Measurement Unit (IMU) attached to a textile band, which communicates via Bluetooth with an external device. The hardware device (MoMo v1 evolved to MoMo v2) and the textile band (textile band v1 to textile band v2) evolve along the years at different times, allowing to improve both patient and clinicians comfort during wrist rigidity evaluation.

The iHandU system includes a wearable device named Motion Mote (MoMo), a Bluetooth-enabled inertial measurement unit (IMU), attached to a textile band, on the palm of the patient’s hand, which communicates via Bluetooth with an external device. Over these years, the system has undergone two main developments concerning the wearable device (see Figure 3): first, the IMU was improved with a smaller design, maintaining all the features, with the aim to obtain a smaller form-factor (MoMo v2). The frequency rate was changed from 42 to 50 Hz due to the higher computational power of the MCU; More recently, the textile band of the iHandU 1.0, 2.0 and 3.0 (textile band v1) was also re-designed with the support of a high-quality national textile company (Petratex®) with whom we have been working since 2015 due to their innovative and patented textile techniques that are of most importance for the development of new prototypes. It allowed to increase the comfort of the wearable device for both patient and clinician. This new band (textile band v2), which is part of the iHandU 4.0, presents some advantages over the previous one, since it was specially designed taking in account all the feedback and contribution of the clinical experts. To summon, it was an iterative development resulting in a slimmer, more comfortable and hypoallergenic textile band that can be placed in the patient’s hand more easily and, therefore, more quickly. Another important advantage is its possibility of hardware part removal for washing, which is of major importance to avoid any contamination.

### 3.3. Software Evolution

**Figure 4:**
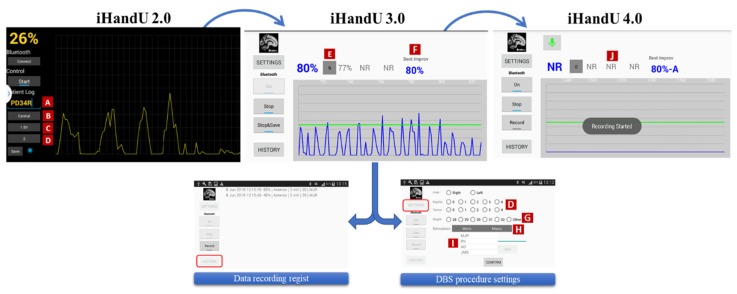
Evolution of the iHandU Android application. All applications were developed to receive the MoMo data and processes it in real time, as well as to record important DBS-related information, using a user-friendly interface. In the iHandU 2.0, the user was able to record the patient ID (A), the anatomical place of stimulation (B), the voltage of stimulation (C) and the rigidity UPDRS (D); the iHandU 3.0, in addition to recording the previous information, also made it possible to collect other relevant information, such as the depth (G) and type of stimulation, the examiner’s name, the tremor UPDRS (D) and the number of cogwheel rigidity artefacts (E). This interface also included a history of stimulations button and the best result was exhibit in the main windows (F). From then on, all data coming from the three axes of the gyroscope and accelerometer sensors were stored in our database. Recently, the iHandU 3.0 was complemented with the voice recording button (J), allowing the recording of all information related with the DBS procedure. This version (iHandU 4.0) is currently being used.

In the first iHandU version, the data visualization and computation of wrist rigidity improvement during DBS was performed through a National Instruments LabView 2014 script [13] on a Laptop. In this version, relevant information, such as the patient ID and stimulation parameters (voltage, depth and anatomical place of stimulation), was recorded manually, while the system’s user provided the classification of rigidity reduction to clinicians. This methodology was disadvantageous, since for most of the surgery procedure, the user was not available to collect all the information. Also, a laptop was not reliable at all to be used in the intra-op environment, due to the space that it occupies, its usability, and even due to security reasons. To overcome these issues, our group have initiated efforts to turn the system more user-friendly and easy to use during BDS surgery: First, we adopted an Android smartphone instead of a laptop. Then, we developed a dedicated application capable to acquire data, visualize and compute the wrist rigidity improvement. This development was an iterative process and we are still adding new features and new characteristic, showing a more versatile and option which is easier to adapt. Figure 4 shows how this application has been evolving along with the iHandU system evolution.

In the iHandU 2.0, a custom-made Android application was developed to receive the MoMo data and process it in real time with a simple user-friendly interface with the capability to collect surgery-related information, such as the patient’s ID, the stimulation voltage and the anatomical place of stimulation (stimulation parameters that varied the most throughout the surgery) and the rigidity UPDRS (Unified Parkinson’s Disease Rating Scale from 1 to 4, where 4 is the more severe case) of the patient, since this version has included the Multi-Model [15].

After testing and retrieve feedback during intra-operatory assessments we understood that some aspects and interface interaction should be changed, so the iHandU 2.0 was updated and the version 3.0 was created. This version as a new interface designed to record more pertinent surgical information, such as the tremor UPDRS, the type of stimulation (micro or macro-stimulation) and the cogwheel artefacts number as shown in Figure 4. This version included both Cogwheel Rigidity Model and 3-Gyroscope-Axis Models, therefore, the application was also designed to record the angular velocity of all gyroscope-cartesian-axes. In addition, the iHandU 3.0 also started to record information from the 3-axes of the accelerometer sensor, that will be used in future mathematical models (we will address this topic in conclusion). Other functionalities of the new interface included the history of stimulations option and the display of the best wrist rigidity improvement [19]. This iHandU application has enriched our database and facilitated the exchange of information between our classification system and the clinicians. However, it was not yet possible for us to record all the information underlying the surgical procedure, such as the stimulation side effects, or the optimal stimulation parameters chosen by clinicians.

**Figure 5:**
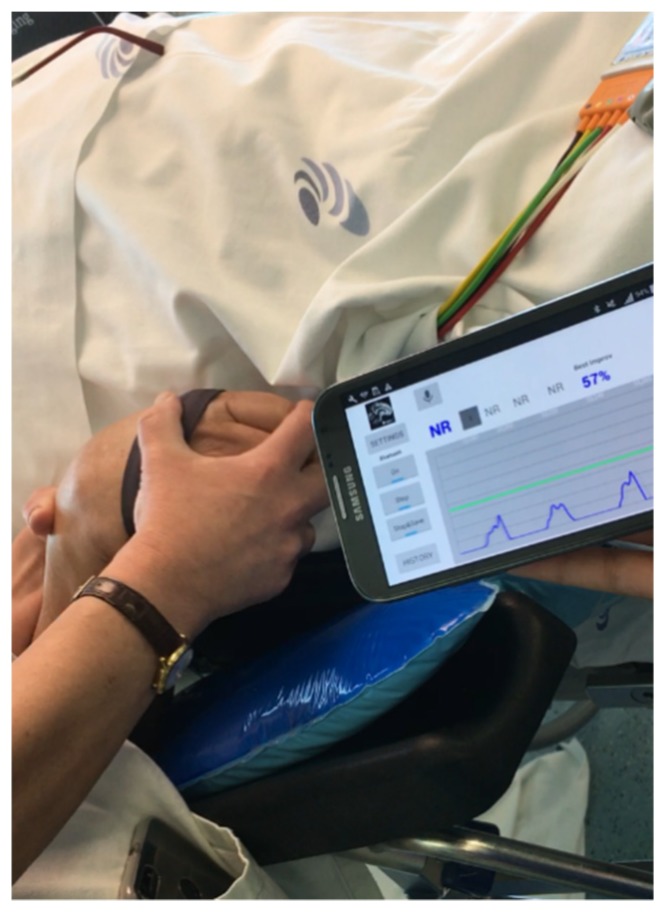
Real-time wrist rigidity improvement classification during the DBS surgery performed using iHandU 4.0 and the most recent wearable device in Centro Hospitalar Universitário São João, E.P.E., Porto, Portugal.

To overcome the lack of this information it was added a voice recording option that enable to record the voice during DBS surgery and be able to have the missing information. This functionality was recently integrated in the iHandU system leading to the iHandU 4.0 version. It also allows the user to record any relevant information related with the DBS procedure and it is currently being used in-surgery (Figure 5).

## 4. Wrist Rigidity Classification Models

**Figure 6:**
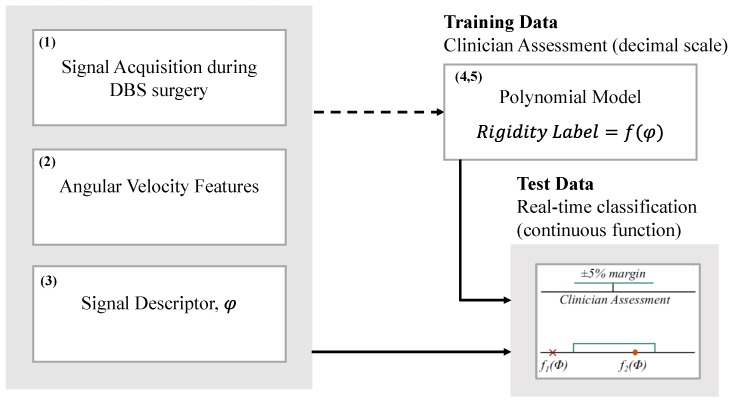
General framework of the iHandU’s wrist rigidity classification model. The dotted arrow indicates that the procedure was done offline, while the continuous line arrow indicates that the processing was done online during surgery.

So far, four iHandU system versions have been developed. In all versions, a rigidity classification model has been developed to assess the wrist rigidity reduction during the DBS procedure, which all of them shared a similar framework of rigidity classification (Figure 6). This framework comprises the following steps: (1) signal is acquired during the DBS surgery for a group of patients, i.e., the training set; (2) features are extracted from the signal, reflecting different rigidity levels; (3) all features are combined in one function, designated as signal descriptor; (4) the average values of the signal descriptors belonging to the same medical label classes of rigidity improvement, ranging from 0 to 80%, are computed; (5) the second-order polynomial function that best model the relationship between the percentage of rigidity improvement (Rigidity Label) and the signal descriptor is estimated; (6) the previous function is used to classify the rigidity improvement of a second group of patients during the DBS surgery, i.e., the test set. A classification is considered accurate if it is within a range of 5% away from the clinician’s classification.

In the first model, the angular velocity during the passive wrist flexion was recorded using the *y*-axis of the gyroscope sensor, from which two features were computed for each 300 samples: the average angular velocity value and the angular velocity peak. The number of samples was defined so as to ensure the presence of a minimum of two to three arches in signal processing. Then, these features were combined using Equation (1). As a result, the polynomial function present in Figure 2 was estimated. This classification model labeled correctly 83.9% of the evaluated signals against the blind agreement of two specialists. The main limitations were found when classifying intermediate levels of wrist rigidity. Our research group has been trying to overcome some limitations of the first model since 2015. Four models were developed so far: The (A) multi-model (MM), the (B) cogwheel rigidity model (CRM), the (C) three-gyroscope-axis model (3GAM) and the (D) reduced model (RM). 

(A) Multi-model: consists of two rigidity models, specific for each baseline wrist rigidity: low or high rigidity states, measured before stimulation. The most appropriate model can then be selected at start-off of the surgery, according to the physician’s initial assessment [15].

(B) Cogwheel rigidity model: The cogwheel rigidity was included in the signal descriptor as a third angular velocity feature [19]. To detect cogwheel rigidity from the angular velocity signal, we used a methodology developed by Costa et al., [13].

(C) Three-gyroscope-axis model: The angular velocity of the three Cartesian axes was considered instead of only the *y*-axis [19] and, finally.

(D) Reduced Model: the number of samples to extract the angular velocity features (angular velocity mean value and angular velocity mean peak) was decreased from 300 to 200 [19].

Since the models were not developed at the same time, different datasets, as well as different iHandU versions were used. Table 4 summarizes the dataset characterization and Table 5 correlates the type of data, features extracted, system version and dataset considered to each wrist rigidity model. The experiment considered in all models during the surgical procedure was similar to that described above.

### 4.1. Signal Processing

In all models, data was acquired and processed in a smartphone. Only the 3-gyroscope-axis model considered data from the 3-axis of the gyroscope sensor. In all other models, only the data of the *y*-axis of the gyroscope sensor was considered. Signal processing was similar to the performed for the first model [13]: First, the raw data was converted to angular velocity, according to the previously presented Equation (2). Then, only its negative values were considered, restricting the analysis to the flexion wrist movement. In the Multi-model, features were extracted for every 300 samples, while in the remaining ones, the features were extracted for every 200 samples. For Multi-model, 3-gyroscope-axis model and Reduced model, two features were considered: average angular velocity and angular velocity peak value. In the 3-GAM, these features were extracted in all Cartesian axes. In the Cogwheel rigidity model, on the other hand, a third feature was considered: the cogwheel artefact number, δ. The angular velocity features were combined by several signal descriptors, aiming to describe the decrease in rigidity. Table 6 summarizes the signal descriptors considered in each wrist rigidity model.

### 4.2. Classification Model

Each signal descriptors present in Table 6 were used to build a wrist rigidity model where the rigidity during the passive wrist flexion was labeled according to the medical expert’s assessment agreement, following a discrete decimal scale, ranging between 0 and 80% of rigidity improvement. We then computed the average values of the signal descriptors belonging to the same medical label classes. Contrary to what was done in the first model, now we only considered the classes 0%, 40%, 50%, 60%, 70%, and 80% of improvement, since the rigidity improvement between 0% and 40% was considered more difficult to discriminate by the clinicians. Finally, the polynomial models that best approximate the relationship between the percentage of rigidity improvement and the signal descriptor were derived. We used a custom-made program running on MathWorks^®^ MATLAB to analyze the training set of each rigidity model. In the Multi-model, the training set was separated in two different clusters: low baseline rigidity (98 signals), which corresponds to UPDRS scores of 1 or 2, and high baseline rigidity (139 signals), in which the patients had an UPDRS of 3. Patients with UPDRS 4 were not found for this work. Each of these subsets were used to obtain the classification model. In all rigidity models, the training errors were estimated using the Leave-one-out cross-validation method with 5000 iterations. The signal descriptors with the lowest training error of each model were tested during DBS surgery. 

### 4.3. Results and Discussion

**Figure 7:**
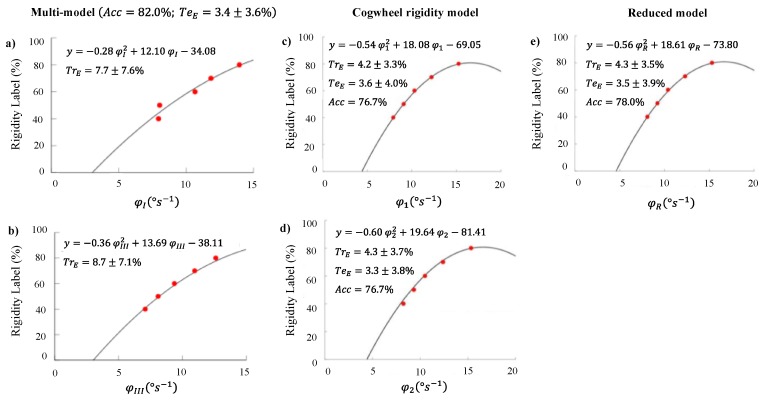
Polynomial models tested during DBS surgery: (**a**) Low baseline rigidity of Multi-model considering the signal descriptor $\varphi_{I}$; (**b**) High baseline rigidity of Multi-model considering the signal descriptor $\varphi_{III}$; (**c**) Cogwheel rigidity model considering the signal descriptor $\varphi_{1}$; (**d**) Cogwheel rigidity model considering the signal descriptor $\varphi_{2}$; (**e**) Reduced model. Adapted from [15,19].

The signal descriptors $\varphi_{I}$ and $\varphi_{III}$ (low and high baseline rigidity models, respectively), $\varphi_{1}$, $\varphi_{2}$ and $\varphi_{R}$ which presented the larger accuracy and the lowest test error (maximum: 3.6 ± 4.0%), leading to the best system performance, as presented in Figure 7, including the accuracy, training and test errors obtained. The remaining signal descriptors of Table 6 were discarded since they exhibited higher training errors. 

From the analysis of this figure, we can conclude a high fitting of the mean values of the signal descriptors in each step of improvement, for all wrist rigidity models. The worse fitting was the one of the Multi-Model of patients initially less rigid (Figure 7a). This could be related to a higher difficulty by the examiner to identify small differences when the rigidity baseline is low. For all rigidity models, approximately the same accuracy was obtained (~80%)—except for the 3-gyroscope-axis that decreased the system performance—supports the reliability of this approach to sustain the examiner’s assessment in intra-operatory conditions, by providing real-time feedback. It also is the same result as the first model, suggesting, since these models were trained and tested for a larger number of patients, that a larger dataset does not compromise the system performance. At the same time, the training error was smaller when we considered a larger dataset. 

The classification error, i.e., the test error, was similar for all models, supporting the idea that the polynomial regression is a good representative model, even when we are comparing a continuous scale to a decimal one. On the other hand, the classification error did not decrease considering the Reduced model compared to the others “single-axis” models, as we expected. However, the decrease of the number of samples to extract the angular velocity features does not compromise system performance. This is a valuable result, since it suggests less time for each intraoperative evaluation, reducing the overall procedure duration. This may bring benefits to the patients, who are having awake brain surgery, decreasing the global risk of surgical infection.

Further, contrary to what was expected, a multi-model approach did not improve the system performance. This proves the limitation of kinematic measures in further distinguishing different wrist rigidity levels since both models lead to similar results, even though differences were evident. Hence, we expected that the cogwheel rigidity, which is considered one major feature in PD rigidity, that highly distinguishes UPDRS sub-scores of 2 and 3, could increase the system performance. However, our results suggest that the cogwheel rigidity does not increase the system’s performance when compared with the first model. In fact, with the methodology used, it is possible to detect cogwheel rigidity even for 80% of rigidity improvement and similar average of these artefacts can be found for intermediate levels of rigidity (see Table 7). This suggests that this angular velocity feature may not play a role in intermediate improvements.

Finally, from the angular velocity of *x* and *z*-axis we may conclude the *z*-axis does not vary with the expert’s classification agreement. The *x*-angular velocity, on the other hand, varied with the wrist rigidity reduction but this gain did not cause any improvement to our system. Therefore, the decrease in system performance may be related to the *x*-axis associated noise. 

## 5. Conclusions and Future Directions

This work reported the iHandU system evolution, from its first proof-of-concept until the most recent version that is being used nowadays. Also, we described the main lessons learned in each iteration, enabling the system improvement and adding relevant scientific knowledge related with wrist rigidity assessment during DBS surgeries, a technique that is being used worldwide. The collected feedback during the system evolution made possible to achieve a more robust, user-friendly and customized system, but also new features and improvements that will be made in the next iteration named iHandU 5.0, furthered explained.

The iHandU is proving its capability to support clinicians showing very good results, as shown before. This system evolution results from a high level of interaction between medicine and engineering area resulting in a persuasive system focused on personalized health with direct impact on healthcare procedures. The evolution of iHandU 1.0 to iHandU 2.0 with the introduction of a Smartphone was crucial to improve the system reliability enabling its adaptation to the clinical environment without any interference with the normal surgical procedure. Another adaptation that has recently improved the clinical assessment was the new textile band (textile band v2) that is not only contributing for a higher comfort of the patient, but mainly in the wrist rigidity assessment method allowing clinicians to easily hold the patients hand without feeling the interference of a wearable device. This contributed for a more standard evaluation assessment and for iHandU it represents a huge evolution in the system adaptation to the clinical environment requirements. Regarding the wrist rigidity classification, it was of the utmost importance to understand and explore if it was possible to obtain better results using more parameters. As stated in previous publications [13,15,19] and in this work, although an effort was made with the development and test of new models it was still not possible to achieve a significant improvement due to the high accuracy already presented by the first model. This fact allows us to conclude that our results showed that y-gyroscope-axis remains the best way to classify the wrist rigidity reduction and also the fact that in all iterations, the y-gyroscope axis showed an accuracy of ~80%, proving the robustness of our model. On the other hand, although the y-Reduced Model (only 200 samples for wrist rigidity improvement classification) does not improve the model overall accuracy, it can be considered as our best result, since it optimizes the signal processing methodology.

In this work we introduced the last version, the iHandU 4.0, currently used in DBS surgeries at São João Hospital in Portugal. This version includes: (1) the textile band v2; (2) the MoMo v2 with a smaller design (i.e., a smaller formfactor); (3) the y-reduced model and (4) the iHandU 4.0 Andorid App, in which a voice recording button was added to record all information related with the DBS procedure.

The authors have in mind that other biomechanical properties could eventually be explored to improve our classification system, derived from the resistive torque, as in [7,8,9,11,12]. However, as was described previously, most setups designed for rigidity quantification are bulky and complex, hence, inappropriate to take into the operating room. Additionally, some of these are not easily available and clinically not applicable [11]. Others still depend on the velocity of the imposed movement [14]. Intermediate improvements of wrist rigidity are often related to false improvements: a decrease in wrist rigidity means less resistive force to imposed movement and, if an expert applies about the same force for each assessment, then higher values of angular velocity will be achieved; nonetheless, if the expert, when feeling less resistance, imposes less force, then no significant change in velocity will occur, thus, no improvement or less than expected is observed. Therefore, the addition, to our system, of force sensors capable of acquiring the force that the examiner imposes in every trial, may lead to the correction of false improvements, therefore enhancing the system’s performance. Adding these sensors, the authors believe that the development of a new model can enable to perceive whether the patient actively participates in the passive flexion, leading to the false detection of improvements. 

To explore these new possibilities and understand how to improve even more the iHandU system in the main three areas (hardware, software and wrist rigidity classification), our research group is already working in the iHandU 5.0 version that will integrate the force sensors integrated in an improved textile band and a new wrist rigidity classification model containing information about the clinician force. Also, this version will contemplate a new redesigned hardware device with Bluetooth Low Energy to reduce power consumption and with a much small form-factor. These new hardware developments are showing that we are able to reduce the device consumption around four times and reduce the size by almost five times when comparing with the existing one. The synergy between the textile band and the hardware is also under development and in a near future the wearable device will contemplate conductive yarns and new sensing features to contribute for an improvement of the rigidity model.

Prospectively, we also intend to design an “intelligent” system, able to refit the polynomial function in each wrist rigidity assessment, since the system classifies every train of 200 samples (4 s), the signal descriptor and the medical label can be added to the training set. It is also important to perform a system validation with a larger number of patients across different clinical centers, to support its use as a standard tool to assist DBS surgery. For this purpose, some connections with international centers is being made to perform a designed study protocol, in which we propose the system to be first used by a top-level experience neurologist, to find the best polynomial model for signal classification, and therefore, be used, in the future, as a learning tool for less experienced DBS specializing neurologists. Also, the iHandU’s usability may be extended to diagnostic and follow-up of PD patients, not only focused on rigidity evaluation, but also other cardinal symptoms, such as tremor and bradykinesia, which are already under development. 

Finally, we foresee the use of iHandU as a supporting tool to evaluate the effectiveness of therapeutic drugs during pharmacological clinical trials procedure to enable the quantification of drug effects, as well as in the clinical response to acute Levodopa challenge test (LDCT), to predict the efficacy of levodopa in the long-term treatment of PD.

Until now, the iHandU system has been used to classify the wrist rigidity improvements of more than 100 patients, corresponding to more than 1600 classifications (107 minutes of data acquisition). A database of wrist rigidity evaluation is being built to be shared as the first database (to the best of our knowledge) of patient inertial data during DBS surgery with a medical label. 

## Figures and Tables

**Table 1:** Summary of methods for rigidity assessment taking in account the method, the body region, the needed parameters, if the system is wireless and if it gives a real-time feedback. The highlighted lines correspond to scientific publications related with the iHandU system—innovative on making use of wireless technology and the only one with real-time feedback. EMG stands for electromyography and MMG for mechanomyogram.

Reference	Method	Body Region	Parameters	Wireless	Real-Time Feedback
Patrick et al. (2001) [6]	EMG Torque-angle	Elbow Wrist	Mechanical impedance	No	No
Shapiro et al. (2007) [7]	EMG Torque-angle	Elbow	EMG, Work	No	No
Mak et al. (2007) [8]	Torque-angle	Trunk	Peak torque	No	No
Endo et al. (2009) [9]	EMG Torque-angle	Elbow	Viscous damping, Elastic stiffness, Difference of Bias	No	No
Levin et al. (2009) [10]	EMG and goniometer	Elbow	EMG	No	No
Park et al. (2011) [11]	Torque-angle	Wrist	Viscous damping, Elastic stiffness, Work, Impulse	No	No
Kwon et al. (2014) [12]	Torque-angle	Wrist	Viscous damping, Elastic stiffness, Work	No	No
Costa et al. (2015) [13]	Angular velocity	Wrist	Average and peak angular velocity	Yes	Yes
Shah et al. (2016) [14]	Acceleration	Wrist	Average acceleration	Yes	No
Assis et al. (2016) [15]	Angular velocity	Wrist	Average and peak angular velocity	Yes	Yes
van den Noort et al. (2017) [16]	Torque-angle Force	Wrist	Elastic stiffness Impulse Work	Yes	No
Angeles et al. (2017) [17]	Angular velocity Force MMG	Elbow Wrist	Mean and standard deviation MMG, Mean force, Mean and standard deviation angular velocity	Yes	No
di Biase et al. (2018) [18]	Angular velocity	Elbow	Smoothness index	Yes	No
Lopes et al. (2019) [19]	Angular velocity	Wrist	Average and peak angular velocity, Cogwheel rigidity	Yes	Yes
Perera et al. (2019) [20]	Force per degree of flexion	Third digit of the hand	Force rate	Yes	No

**Table 2:** Discrimination power between rigid and non-rigid states using the computed angular velocity (in °s^−1^) features (*p*-value < 0.05). Abbreviations: Std: Standard deviation. Extracted from [13].

Feature	Rigid State		Non-Rigid State		*p*-Value
	Mean	Std	Mean	Std	
$\mu_{\omega_{y}}$	3.33	0.58	5.62	1.51	0.034
$\mu_{p_{y}}$	12.9	3.13	29.9	6.60	0.029
$\varphi_{y}$	6.55	1.22	11.3	3.07	0.027

**Table 3:** iHandU system evaluation. After the first iHandU system (iHandU 1.0) development, three more versions were built: iHandU 2.0, 3.0 and 4.0. These system versions differ in hardware, software and rigidity classification model. Each iteration was developed with the aim of overcoming some of the limitations underlying the previous version.

Year	iHandU System	Hardware	Software	Rigidity Classification Model
2015	iHandU 1.0 [13]	• Textile band v1 • MoMo v1 • Y-Gyroscope-Axis • Laptop (Intel Core i7 CPU @ 2.70 GHz)	iHandU 1.0 Android App: • Signal visualization • Rigidity reduction classification	y-gyroscope model
2016	iHandU 2.0 [15]	• Textile band v1 • MoMo v2 • y-Gyroscope-Axis • Samsung Galaxy Note II Quad Core 1.6 GHz	• iHandU 1.0 Android App • Patient ID • Stimulation voltage • Place of stimulation • Rigidity UPDRS	Multi-model
2018	iHandU 3.0 [19]	• Textile band v1 • MoMo v2 • 3-Axis-Gyroscope • 3-Axis Accelerometer • Samsung Galaxy Note II Quad Core 1.6 GHz	• iHandU 2.0 Android App • Examiner’s name • Number of cogwheel artefacts • Best rigidity improvement • History of stimulations	• Cogwheel Rigidity Model • 3-Gyroscope-Axis Model • y-Reduced Model
Present	iHandU 4.0	• Textile Band v2 • MoMo v2 • Gyroscope • 3-Axis Accelerometer • Samsung Galaxy Note II Quad Core 1.6 GHz	• iHandU 3.0 Android App • Voice recording	y-Reduced Model

**Table 4:** Dataset characterization: number of patients (signals) submitted to DBS surgery, whose reduction of wrist rigidity was also evaluated with the iHandU system.

Dataset	Training Set	Test Set
Dataset I	6 (48 signals)	4 (156 signals)
Dataset II	17 (237 signals)	2 (38 signals)
Dataset III	22 (118 signals)	8 (150 signals)
Total	45 (403 signals)	14 (344 signals)

**Table 5:** Wrist rigidity models’ evaluation. Symbols: $\mu_{\omega }$—mean value of angular velocity; $\mu_{p}$—mean peak value of angular velocity; δ—number of cogwheel rigidity artefacts.

Rigidity Model	Reference	Data	Features	iHandU Version	Dataset
First model	[13]	y-gyroscope-axis	$\mu_{\omega }$, $\mu_{p}$	1.0	Dataset I
Multi-model	[15]	y-gyroscope-axis	$\mu_{\omega }$, $\mu_{p}$	2.0	Dataset II
Cogwheel rigidity model	[19]	y-gyroscope-axis	$\mu_{\omega }$, $\mu_{p},\;\delta$	3.0	Dataset III
3-Gyroscope-Axis model	[19]	3-gyroscope-axis	$\mu_{\omega }$, $\mu_{p}$	3.0	Dataset III
y-Reduced model	[19]	y-gyroscope-axis	$\mu_{\omega }$, $\mu_{p}$	3.0	Dataset III

**Table 6:** Signal descriptors computed for each rigidity model. A. $\varphi_{y}=\sqrt{\mu_{\omega_{y}}.\mu_{p_{y}}}$; B. $\omega_{n2}=\sqrt{\underset{i=\left\{x,y\right\}}{\sum }\omega_{i}^{2}}$; C. $\omega_{n3}=\sqrt{\underset{i=\left\{x,y,z\right\}}{\sum }\omega_{i}^{2}}$.

Multi-Model	3-Gyroscope-Axis Model	Cogwheel Rigidity Model	Reduced Model
$\varphi_{I}=\varphi_{y}$ ^A^	$\varphi_{\mathrm{norm}2}=\sqrt{\mu_{\omega_{n2}}.\mu_{p_{n2}}}$ ^B^	$\varphi_{1}=\sqrt{\varphi_{y}^{2}-\delta }$	$\varphi_{R}=\varphi_{y}$
$\varphi_{II}=\min\left(\mu_{\omega_{y}},\mu_{p_{y}}\right)$	$\varphi_{\mathrm{norm}3}=\sqrt{\mu_{\omega_{n3}}.\mu_{p_{n3}}}$ ^C^	$\varphi_{2}=\sqrt{\varphi_{y}^{2}+\delta }$	
$\varphi_{III}=\frac{\mu_{\omega_{y}}}{2}\;log_{2}\left(\frac{\mu_{\omega_{y}}+\mu_{p_{y}}}{\mu_{\omega_{y}}}\right)+\frac{\mu_{p_{y}}}{2}\;log_{2}\left(\frac{\mu_{\omega_{y}}+\mu_{p_{y}}}{\mu_{p_{y}}}\right)$	$\varphi_{xy}=\sqrt{\underset{i=\left\{x,y\right\}}{\sum }\mu_{\omega_{i}}.\mu_{p_{y}}}$	$\varphi_{3}=\frac{\varphi_{y}-\sqrt{\delta }}{2}$	
$\varphi_{IV}=\varphi_{y}\exp\left(-\frac{\log{\left(\frac{\mu_{p_{y}}}{\mu_{\omega_{y}}}\right)}^{2}}{2\sigma^{2}}\right)$	$\varphi_{xyz}=\sqrt{\underset{i=\left\{x,y,z\right\}}{\sum }\mu_{\omega_{i}}.\mu_{p_{y}}}$	$\varphi_{4}=\frac{\varphi_{y}+\sqrt{\delta }}{2}$	
	$\bar{\varphi }=\frac{1}{3}\left[\underset{i=\left\{x,y,z\right\}}{\sum }\mu_{\omega_{i}}.\mu_{p_{y}}\right]$	$\varphi_{5}=\varphi_{y}-\delta$	
		$\varphi_{6}=\varphi_{y}+\delta$	
		$\varphi_{7}=\sqrt{\mu_{\omega_{y}}.\left(\mu_{p_{y}}-\delta \right)}$	
		$\varphi_{8}=\sqrt{\mu_{\omega_{y}}.\left(\mu_{p_{y}}+\delta \right)}$	

**Table 7:** Average of the number of cogwheel rigidity artefacts ($\bar{\delta })$ and standard deviation (Δδ) for each medical label.

Medical Label	$\bar{\delta }\;\pm \;\Delta \delta$
40%	2.6 ± 1.6
50%	2.2 ± 1.7
60%	2.2 ± 1.6
70%	2.0 ± 1.6
80%	1.2 ± 1.4
